# Content and construct validation of language acquisition enunciative signs in the second year of life

**DOI:** 10.1590/2317-1782/20202020252

**Published:** 2021-12-20

**Authors:** Isabela de Moraes Fattore, Anaelena Bragança de Moraes, Anelise Henrich Crestani, Adriano Mendonça Souza, Ana Paula Ramos de Souza

**Affiliations:** 1 Universidade Federal de Santa Maria – UFSM - Santa Maria (RS), Brasil.

**Keywords:** Language Acquisition, Child Development, Test Validation, Risk Factors, Early Intervention

## Abstract

**Purpose:**

To analyze the content and construct validity of enunciative signs of language acquisition for children aged 13 to 24 months.

**Methods:**

The signs created were subjected to an assessment of clarity and relevance by six expert judges in language acquisition from an enunciative perspective. Based on their judgment, an experimental version was produced that was applied to the analysis of videos of mother-baby / examiner interactions, lasting 15 minutes, of 77 mother-baby dyads aged 13 to 18 months and 89 dyads in the age range aged between 19 and 24 months. The validity of reliability and internal consistency was performed by analyzing 10% of the sample by two speech therapists. The construct validation was carried out by the factorial analysis carried out on the total sample. The data were analyzed in Statistica 9.1 and PASW 17.0.

**Results:**

All signs were considered clear and relevant by the expert judges. The reliability analysis showed an almost perfect agreement (0.8 ≤ Kappa ≥ 1.0) for most signs. The internal consistency for Phase 3 showed alpha = 0.771 considered high and Phase 4 presented alpha = 0.917 bordering on very high. The factor analysis of phase 3 revealed 2 factors, explaining 59.1% of the total variance and phase 4 revealed 1 factor, explaining 75.7%.

**Conclusion:**

Content and construct validity were observed for five of the twelve signs in Phase 3 and for all signs in Phase 4.

## INTRODUCTION

Enunciative Signs of Language Acquisition (ESLA), a screening instrument whose content and construct validation process for the first year of life has already been published^(1,2)^, relies on the theoretical perspective that contributions from both the baby and the adult, which support babies enunciatively, are fundamental in the process of language acquisition^(3)^. From this perspective, dialogue is the unit of analysis in the language assessment process^(4,5)^ that allows verification not only of whether children are progressively mastering the grammar of the language to which they are exposed, but also of whether the environment provides them with conditions of insertion into language functioning. In this sense, the ESLA differ from traditional screening instruments such as DENVER II, which is used in the Brazilian reality and focuses on children's productions without analyzing the environmental context, and whose sensitivity and specificity in the first year of life is questioned^(6)^. In addition, some studies indicate a lack of validated tests for the Brazilian population in this age group^(7,8)^.

The ESLA follows the indicative paradigm of analysis present in other instruments that assess developmental risk^(9)^ related to the process of language acquisition^(10)^. In this sense, signs are taken as clues or indications of the process. Thus, the presence of signs indicates that the acquisition process is occurring as expected for the age group^(1,2)^, considering the logical processes of emergence of enunciative mechanisms^(3)^, whereas their absence indicates that the process may be at risk for an outcome of delayed language acquisition^(1,2)^ from the perspective of the emergence of a speaking individual^(11)^.

It is known that the validation of an instrument is essential so that it can be applied reliability and credibility^(12)^. The validation will show whether the instrument measures what it proposes to measure, and whether the abstract concepts idealized for the instrument have become measurable indicators of the phenomenon of interest. In this process, analysis by experts is required to identify whether the items are understandable to the professional who will use them (clarity), as well as their relevance for measuring the phenomenon proposed for evaluation^(13)^. Also, within the scope of content validation, the use of the instrument by different evaluators or in the test and retest of the same group of individuals needs to demonstrate reliability and reliability^(14)^, based on stable and consistent responses.

Construct validation, which is the next step after content validation, is essential to verify whether the instrument separates groups in a broader sample considering the investigated characteristics, showing that its assessment items discriminate these characteristics, thus generating accurate, valid and interpretable data^(13,14,15)^.

Considering these assumptions, this article aims to analyze the content and construct validation of the ESLA screen instrument for children aged 13 to 24 months.

## METHODS

This study was conducted according to the mandatory ethical standards for research on human beings – Resolution 466/12 of the Brazilian National Health Council (CNS), and the project was approved by the Ethics Committee of the aforementioned Institution under protocol no. 18608413.4.0000.5346. The examiners signed a Non-disclosure Agreement Form and the parents and/or legal guardians of the babies signed an Informed Consent Form prior to study commencement, thus ensuring the confidentiality of the data and the privacy of the participants.

The study addresses the content and construct validation of a language assessment instrument for the second year of life called Enunciative Signs of Language Acquisition (ESLA). The ESLA comprises two phases: Phase 3 - corresponding to the enunciative signs relevant to the 13-18 month age group, and Phase 4 - corresponding to the signs relevant to the 19-24 month age group.

An initial version of the two phases of the instrument was proposed considering the enunciative mechanisms and strategies^(3)^ and the clinical experience with babies who present delayed language acquisition. The prepared signs were analyzed by six experts in language acquisition from an enunciative perspective and, after their contributions to the improvement of these signs, they were assessed by the same examiners with respect to clarity and relevance. To this end, a 5-point Likert scale was used for each proposed sign, as follows: 1- strongly disagree, 2- partially disagree, 3- neither agree, nor disagree, 4- partially agree, or 5- strongly agree. Signs that reached ≥70%^(16)^, assigned to items 4- partially agree and/or 5- strongly agree, remained in the experimental version of the instruments.

Analysis of the mother-baby interactions was based on 15-min videos of interactions between 166 babies aged 13 to 24 months and their mothers. These dyads were recruited at a Basic Health Unit located close to the Institution where the study weas conducted. The videos were recorded during the Neonatal Heel Prick test, and also served as part of the screening process for the babies, who also underwent an ear test and were referred to assessment of the cortical potential to eliminate babies with any type of hearing loss. An interdisciplinary team composed of speech-language pathologists, psychologists, physiotherapists, and occupational therapists assessed the babies, and only those born at term and without physical, auditory and visual impairment were included in the study. In addition to the aforementioned objective tests, the babies were observed for psychomotor, physical and intellectual development by this team. In addition, mothers could not have evident psychiatric disorders and, to ensure that, they were analyzed by psychologists in an initial interview that addressed obstetric, sociodemographic and psychosocial aspects of both babies and their families. Thus, babies who were premature, presented neurological impairment, malformations and syndromes, and who were hearing- or visually-impaired were excluded from the study. If there were any doubts regarding the mother and/or baby, they were referred for more detailed evaluations and discarded from the study.

Filming occurred in a comfortable environment, with adequate lighting and ventilation, with the babies being properly cleaned and fed, without pain or discomfort. During filming, a toy box with miniatures was made available for exploration by the mother and child, who remained on an EVA mat. Two digital cameras were used: one placed on a tripod two meters away caught the reflection of the mother and the baby on a mirror placed by the wall behind the mat, and another placed a meter away filmed the dyad from a lateral angle. For the first 10 min, only the interactions between mothers and babies were filmed. For the last 5 min, the researcher participated by interacting with the baby and the mother to observe signs that involved the baby’s interaction with an interlocutor different from the mother. In addition, to researcher made requests in the dialogue that favored the emergence of protowords and words, when these did not appear in the interaction with the mother.

After collection, the footage was stored in an HD so that the researchers could watch it and analyze the signs whose responses were dichotomous, such as yes for the presence of the sign, and no for the absence of the sign. In the case of signs referring to the adult's actions that dealt with more than one adult action (Sign 7 of Phase 3 and Sign 5 of Phase 4), they were considered present only when the actions were performed. For example, if the mother provided non-attuned interpretations to what the child clearly manifested (Sign 7, Phase 3), the sign would be absent. In the case of Sign 5 of Phase 4, if the mother corrected the child's speech, in any of the three options observed (neutral request for repair, correct repetition of the child's speech, or offer of the correct item), the sign was considered present.

To verify the reliability and thus analyze the applicability and stability of the instrument, the interrater method was used, in which two speech-language pathologists analyzed the videos of 10% of the sample individually, and the results of their analysis were compared to determine the agreement between their answers, calculated by the Kappa coefficient. When the responses were dichotomous, the Kuder-Richardson (K-R) test was used to analyze the internal consistency.

Then the signs were applied to the entire sample. When any of the signs were absent from the 10% analysis and this absence was confirmed in the analysis of the total study sample, it would be eliminated from the factor analysis

From the instrumental version obtained with this analysis of reliability and internal consistency, the final sample had at least 10 dyads for each sign to be observed to generate factor solutions in the multivariate analysis^(13,14,15)^, considering a significance level of 5%. The sample considered at this stage was 77 dyads for Phase 3 and 89 dyads for Phase 4, as previously mentioned. For factor analysis, both the Kaiser-Meyer-Olkin (KMO) coefficient and the Bartlett's test of sphericity were used.

The collected data were stored in an Excel spreadsheet and converted to STATISTICA 9.1, PASW 17.0 and R software. Quantitative data analysis was performed through descriptive and inferential statistics using specific tests for each step, as previously mentioned.

## RESULTS

Chart 1 shows the Enunciative Signs of Language Acquisition (ESLA) constructed by the researchers and presented to expert judges for content analysis.

**Chart 1:** Initial version of the Enunciative Signs of Language Acquisition (ESLA) instrument

Phase 3	*Description*
S1	The child spontaneously and intelligibly names to the adult interlocutor objects that are absent in the context.
S2	The child spontaneously, but not intelligibly names to the adult interlocutor objects that are absent in the context, seeking in prosody a way to be understood.
S3	The child spontaneously and intelligibly names to the adult interlocutor objects, people, and actions that are present in the enunciative context.
S4	The child makes gestures trying to make themselves understood when the adult interlocutor does not understand them.
S5	The child repeats the speech of the adult interlocutor as a way to organize or reorganize their enunciation, for example, improving the syntactic or phonological form, or the choice of the lexical item, or even prosodically accentuating some item.
S6	The child converses with different adult interlocutors (father, mother, examiner).
S7	The adult interlocutor attributes a possible meaning to the child's verbal productions in a tuned way.
Phase 4	*Description*
S1	The child asks for objects and/or asks the adult interlocutor for clarification, marking their position as speaker.
S2	The child uses different phonemic forms to convey different meanings in their enunciation (at least two modes and two points of articulation in mono- or dissyllable productions, e.g., /ma/, /da/, /ta/, /na/, /pa/, /ba/, etc.)
S3	The child uses different forms (words) to convey different meanings in their enunciation
S4	The child combines words, directly or inversely, to convey different meanings.
S5	When the child presents verbal productions that are different from adult speech, the adult interlocutor reacts by making a neutral repair request, correctly repeating the child's speech, or offering a more adequate word.
S6	The child makes comments or qualifications of situations (e.g., it's heavy, good, bad, ok, here, there).
S7	The child demonstrates to reflect on the events through the production of statements with more than one word (e.g., “I don't know if he fell”; “Is he he coming?”)
S8	The child reformulates what they have said when the adult interlocutor does not understanding them and mark themselves
S9	When you do not understand the interlocutor's utterance
S10	The child discursively marks themselves as an “I” (using the pronoun or inflection of the verb).
S11	The child is marked by nominal or pronominal forms other than the “I” in the speech (e.g., we, yours, mine, their own name)
S12	The child inserts a previous saying (theirs or of others) or projects a future saying

Table 1 shows the results of the analysis of clarity and relevance carried out by the expert judges.

**Table 1:** Analysis of clarity and relevance of the signs of Phases 3 and 4 of the ESLA by expert judges

**Signs**	**Clarity Analysis**								**Pertinence Analysis**							
**Phase 3**	J1	J2	J3	J4	J5	J6	%SA	%PC+SA	J1	J2	J3	J4	J5	J6	%SA	%PC+SA
**S1**	4	5	5	4	5	5	66.7	100	4	5	5	5	5	5	83.3	100
**S2**	5	5	5	4	5	5	83.3	100	4	5	5	5	5	5	83.3	100
**S3**	4	5	5	4	5	5	66.7	100	4	5	5	5	5	5	83.3	100
**S4**	5	5	5	5	5	5	100	100	5	5	5	5	5	5	100	100
**S5**	4	5	5	5	5	5	83.3	100	4	5	5	5	5	4	66.7	100
**S6**	5	5	5	5	5	5	100.0	100	5	5	5	5	5	5	100	100
**S7**	4	5	4	4	4	5	33.3	100	4	5	5	5	5	5	83.3	100
**Fase 4**	5	5	5	3	5	5	83.3	83.3	5	5	5	5	5	5	100	100
**S1**	5	5	4	5	5	5	83.3	100	5	5	4	5	5	5	100	100
**S2**	5	5	5	5	5	5	100	100	5	5	5	5	5	5	100	100
**S3**	5	5	5	4	5	5	83.3	100	5	5	5	5	5	5	100	100
**S4**	5	5	5	5	5	5	100	100	5	5	5	5	5	1	83.3	83.3
**S5**	4	5	5	4	5	5	66.7	100	4	5	5	5	5	5	83.3	100
**S6**	5	5	5	4	5	5	83.3	100	4	5	5	5	5	5	83.3	100
**S7**	6	5	5	4	5	5	83.3	100	4	5	5	5	5	5	83.3	100
**S8**	5	5	5	5	5	5	100	100	5	5	5	5	5	5	100	100
**S9**	5	5	5	5	5	5	100	100	5	5	5	5	5	1	83.3	83.3
**S10**	5	5	4	5	5	5	83.33	100	5	5	5	5	5	5	100	100
**S11**	5	5	5	1	5	5	83.33	83.33	5	5	5	4	5	5	83.3	100
**S12**	5	5	5	3	4	5	66.7	83.33	5	5	5	5	5	5	100	100

Caption: J = Judge; %PC = percentage of partial agreement; %SA = percentage of strong agreement; S = sign; 1 = strongly disagree; 3 = neither agree, nor disagree; 4 = partially agree; 5 = strongly agree

The initial criterion for the permanence of a sign came from the percentage of judges who partially and/or strongly agreed with each sign. Percentages of agreement ≤70%^(16)^ were evaluated for their alteration or exclusion by the researchers. As it can be seen in Table 1, there was no percentage lower than 83.3% when adding levels 4 (partial agreement) and 5 (strong agreement) of the Likert scale, both in terms of clarity and relevance. From this analysis, it was possible to build an experimental version of the ESLA, summarized in Chart 2.

**Chart 2:** ESLA experimental version

Phase 3	*Description*
S1	The child spontaneously and intelligibly names to the adult interlocutor objects that are absent in the context.
S2	The child spontaneously, but not intelligibly names to the adult interlocutor objects that are absent in the context, seeking in prosody a way to be understood.
S3	The child spontaneously and intelligibly names to the adult interlocutor objects, people, and actions that are present in the enunciative context.
S4	The child makes gestures trying to make themselves understood when the adult interlocutor does not understand them.
S5	The child repeats the speech of the adult interlocutor as a way to organize or reorganize their enunciation, for example, by improving the syntactic or phonological form, or the choice of the lexical item, or even prosodically accentuating some item.
S6	The child converses with different adult interlocutors (father, mother, examiner).
S7	The adult interlocutor attributes a possible meaning to the child's verbal productions in a tuned way.
Phase 4	*Description*
S1	The child asks for objects and/or asks the adult interlocutor for clarification, marking his position as speaker.
S2	The child uses different phonemic forms to convey different meanings in their enunciation (at least two articulatory modes and points of articulation, e.g., /ma/, /da/, /ta/, /na/, /pa/, /ba/)
S3	The child uses different forms (words) to convey different meanings in their enunciation (presents lexical variety)
S4	The child combines words, directly or inversely, to convey different meanings.
S5	When the child presents verbal productions that are different from adult speech, the adult interlocutor reacts by making a neutral repair request or correctly repeating the child's speech.

Caption: S = sign. Source: The authors

As for the instrument reliability, in the attribution of signs in the analysis of the videos conducted by two judges for Phase 3 (13 to 17 months) and Phase 4 (18 to 24 months), the results obtained are shown in Table 2. In Phase 4, signs 6, 7, 8, 9, 10, 11 and 12 were not found in the 10% of the sample or in the total sample, and were then eliminated. Therefore, Table 2 presents only the concordance of signs 1 to 7 in Phase 3 and 1 to 5 in Phase 4, which were the signs found by the judges in both analyzed samples.

**Table 2:** Interrater agreement for Phases 3 and 4 of the ESLA

**Signs ESLAPhase 3**	**% agreement**	**Kappa Coefficient**	*p*-value*
S1	100	1.00	-
S2	98.7	-	-
S3	88.3	0.746	-
S4	72.7	0.357	0.001
S5	92.2	0.834	-
S6	98.7	0.971	-
S7	92.2	0.798	-
**Signs ESLA Phase 4**			
S1	96.6	0.916	0.000
S2	98.9	0.967	0.000
S3	98.9	0.970	0.000
S4	97.8	0.954	0.000
S5	97.8	0.947	0.000

* Significant by the Kappa agreement analysis at a significance level of 5%

Caption: ESLA = Enunciative Signs of Language Acquisition; % = percentage

In Phase 3, Kappa was not calculated for Sign 2 because it did not show variability in the evaluation of signs by at least one of the judges. Signs 3 and 7 showed substantial agreement (0.6 ≤ Kappa ≥ 0.79), whereas Sign 4 showed substantially weak agreement (0.2 ≤ Kappa ≥ 0.3). Interrater agreement was almost perfect (0.8 ≤ Kappa ≥ 1.0) for Signs 1, 5 and 6 in Phase 3 and for all signs in Phase 4.

The internal consistency method was applied to both phases of the instrument after analyzing the agreement in relation to the signs. The Cronbach's alpha coefficient was used in the total sample, that is, Phase 3 = 77 dyads and Phase 4 = 89 dyads, analyzing each sign individually. For Phases 3 and 4, *α*=0.771 and *α*=0.917 were obtained.

**Figure 1:**
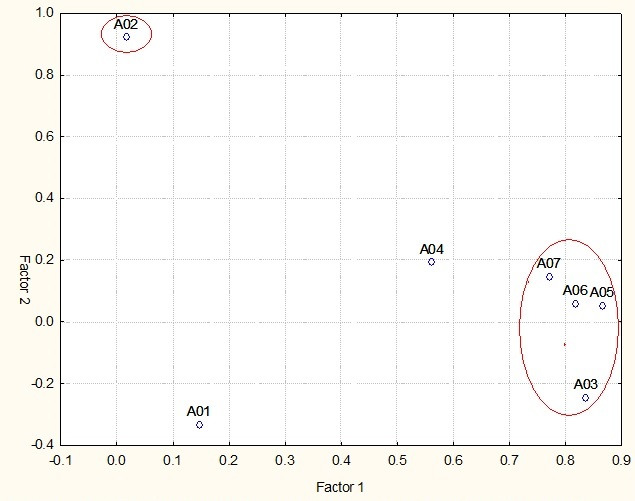
Phase 3- Signals distribution- factore 1 signals 5,3,6,7; factor 2 -signal 2

Source: Multivariate analysis. Caption: A = Signal

Next, factor analysis was performed for Phase 3, as illustrated in Figure 1.

The Kaiser-Meyer-Olkin coefficient (KMO=0.78) was used as a prerequisite for carrying out the factor analysis of the instrument in Phase 3 (13 to 17 months), which is considered moderate to indicate the adequacy of the instrument to the factor analysis, that is, the factor analysis is appropriate. The Bartlett's test of sphericity (159.7; *p*<0.01), whose significance indicates that the variables are correlated with each other, was also applied. Thus, the factor analysis of Phase 3 revealed the existence of 2 factors, which explained 59.1% of the total variance. The analysis with Varimax rotation showed factor 1 with four items and factor 2 with one item. When performing the factor analysis with Varimax rotation, it was observed that factor 1 had four variables of greater relevance, in the following order: Signs 5, 3, 6 and 7 and factor 2 presented one variable of greater relevance, Sign 2.

Factor 1, determined by an eigenvalue λ=3.1 and explaining 46.6% of the variance, represents “the child supported by the adult and with possibilities for progression in lexical acquisition”. Factor 2, determined by an eigenvalue λ=1.1 and explaining 15.5% of the variance, represents “the communicative intention is a factor for the differential diagnosis”.

The factor analysis of Phase 4 revealed the existence of a single factor, which explained 75.7% of the variance. The analysis without Varimax Rotation – since there is only one factor, showed factor 1 with five items in the following order of relevance: Sign 3 (0.938) – The child uses different forms (words) to convey different meanings in their enunciation; Sign 5 (0.870) – When the child presents verbal productions that are different from adult speech, the adult interlocutor reacts by making a neutral repair request, correctly repeating the child's speech, or offering the correct lexical item; Sign 1 – (0.864) – The child requests objects and/or asks the adult interlocutor for clarification, marking their position as speaker; Sign 2 (0.852) - The child uses different phonemic forms to convey different meanings in their enunciation (at least two points of articulation – labial and alveolar, and at least two distinct consonant sound classes –nasal and plosive, e.g., /ma/, /da/, /pa/, etc.); Sign 4 (0.821) –The child combines words in a direct or inverse way to convey different meanings.

## DISCUSSION

Considering the analysis by experts and the interrater agreement, there was a reasonable approval of the instrument. The only sign that showed substantially weak agreement (0.2 ≤ Kappa ≥ 0.3) between the evaluators was Sign 4 of Phase 3. The factor analysis showed that this sign was not included in any of the two factors found in Phase 3 of the instrument, and it was not correlated with any of the other signs, although it was considered clear and pertinent by the experts.

In the factor analysis, Sign 5 had a greater weight in factor 1, which demonstrates the importance of enunciative support in the language acquisition process^(3)^, that is, if the child uses the interlocutor for grammatical construction. This enunciative support is called scaffolding in the international literature^(16)^.

In Phase 3, it also is clear that the agreement between the judges in the application of the signs, given by the Kappa coefficient, indicates excellent agreement for Signs 1, 5, 6, and 7; sufficiently good agreement for Sign 3; and poor agreement for Sign 4, which is confirmed by the absence of the discriminatory force of this sign in the factor analysis and the greater distinctive power of the other four signs.

As for Sign 2 of Phase 3, the presence of this sign in the evaluated children predicts that they may be using prosodic resources to escape from a lesser domain of articulatory gestures. If together with this sign, the absence of Sign 2 in Phase 4 is evident, one can be alert to a risk for the vocal realization of the tongue in Benvenistian terms, which should be an object of observation during the evolution from babbling for the protowords - basic aspects in the organization of the speech praxis necessary for the initial production of the words of a language. A study on language acquisition indicates the relationship between speech and body gesture^(17)^, which allows us to emphasize that Sign 2 of Phase 3 can be taken as evidence of commitment to make oneself understood by the interlocutor, especially if it is present together with intelligible naming in the same phase (Signs 1 and 3) and with the presence of Sign 2 of Phase 4.

As for internal consistency, *α*=0.771 was observed in Phase 3, which suggests that the signs presented do not overlap in the evaluation. This reliability measure indicates the degree of correlation between the items, representing a measure of reliability. Thus, it can be said that the items are correlated with each other, but they are not measuring the same thing, considering the alpha value found. Differently from what was evidenced in Phase 4, the presence of 2 factors was observed in Phase 3.

Factor 1 was composed of Signs 5, 3, 6, and 7, in order of relevance. Signs 5, 3, and 6 are expected in the course of a typical language acquisition considering that the child would be able to use their lexical knowledge, improving it with different interlocutors. Sign 7 demonstrates the role played by the adult in supporting the child in dialogue in order to keep them engaged in this construction with the other. The adult gives meaning to the child's words, even if the form is not easily understandable, but it can be understood from the context. When the signs of factor 1 are absent, a set of enunciative conditions is evidenced, at an early stage, that shows that the child has not entered the second enunciative mechanism, which is related to the possibility of going from the reference shown to what is spoken, that is, they may show that the child may have their position as a speaker shaken in the social environment, with doubts regarding the assumption of being a speaker of the language.

Factor 2 in Phase 3, composed only of Sign 2, suggests the difficulties of speech intelligibility of a child compensated by prosody, that is, that the vocal performance of the language^(3)^ may be impoverished and that the clinical outcome could be, or not, a speech pathology after being diagnosed later. Again, it is worth noting that studies with clinical outcomes that may, or may not, confirm this hypothesis will be possible after the validation of these signs. This difficulty evidenced between 18 and 24 months of age can lead to adults with difficulties in recognizing the sign, one of the risk indicators found by Verly and Freire^(11)^ when analyzing cases of language acquisition delay.

In Phase 4, the interrater agreement was excellent for all proposed items. In the internal consistency, performed by Cronbach's alpha coefficient, the alpha in Phase 4 was slightly above 0.90, indicating possible redundancy or duplication of items regarding what they are measuring, that is, all signs have the same diagnostic value. This finding was confirmed in the factor analysis, in which a single factor was found in this phase.

In an enunciative reading, the more capable the child is of establishing co-reference with the listener, through varied lexical items, the more the child demonstrates semiotic domain of the language, as well as expansion of strategies of the second enunciative mechanism^(3)^. In addition to presenting an expanded lexicon, the child begins to enunciate in a more complex way during interlocution. Thus, the larger the lexicon, the greater the facilities in linguistic comprehension and production, which Silva^(3)^ stated that is significantly expanded in functions such as interrogation and subpoena, as well as in taking different discursive positions in the third enunciative mechanism.

In Phase 4, it is already evident whether there are repairs or correct repetition of the child's speech by the adult, as described in Sign 5. The relevance of Sign 5 (0.870) relates to how much the adult demonstrates to be or not attentive to what the child produces and how far this production distances itself from the adult form, and may receive repairs from the adult.

Sign 3 of Phase 4 (0.938), considered the most relevant of all those comprising the factor, shows that the child is using the lexical variety that is appropriate for their age group, not restricting themselves to repeated lexical items or neutral forms such as demonstrative pronouns. From the protowords, the child starts to produce the first words, which already show a variation of accentuation and with the function of communicating something. Given the lexical domain that the child acquires, especially of verbs, the appearance of sentences becomes frequent at a slightly older age^(3)^.

Also, in Phase 4, Sign 1 (0.864) already indicates the child's presence in the third enunciative mechanism^(3)^, in which the individual is established in the speech-language, that is, the child is already inserted in the apparatus of functions, taking the initiative to intimate and interrogate, marking their place as an individual in the dialogue with the other. In addition to being a speaker of the language, they mark themselves in different ways in speech^(18)^.

As for Sign 2 of Phase 4 (0.852), it is related to the greater sensitivity to detect early absence of risk for the vocal performance of the tongue, that is, the performance of the articulatory gesture, as already discussed. The child seeks the capacity for phonetic variation in performing the articulatory gesture. Therefore, it has been proposed that the signs allow the child to follow the course of production of oral praxis and take some measures of family guidance and/or intervention in time in order to take advantage of brain plasticity, since the earlier the intervention, the better the chances of overcoming symptoms. This has led to neuroscientific studies addressing early sensory processing in babies^(19)^, as well as socioeconomic factors that can interfere with brain development, especially parental education^(20)^. It is possible to use the enunciative support^(3-5)^ and prosodic strategies of musical anchoring of body and oral gestures to facilitate the evolution of babies at psychic risk and the acquisition of language^(21)^. Music was especially important in the clinical case report of the treatment of speech apraxia in a 5-year-old child. It allowed a significant approach in the articulation between listening and speaking^(22)^. It should be noted, however, that risk is not a diagnosis and it must be taken in a way to create opportunities for promotional acquisition strategies without creating ghosts in the parental imagination. Therefore, we believe in the use of music as an interesting intervention strategy for children aged 18-24 months.

One aspect to be highlighted is that both the baby signs and the signs of the adult interlocutor of the baby, in the case of this study, the biological mother has always shown potency in identifying differences in the sample. This indicates that enunciative support^(3)^ is crucial in the process of child language acquisition, given the intersubjective nature of language functioning^(18)^.

Limitations to this study include the variation in the return of cases for evaluation continuity, which restricted the obtaining of a larger sample.

It is important to emphasize that the ESLA instrument is still far from being validated, as it is necessary to establish the criteria for the emergence of signs at each age group, as well as to carry out population cohort studies, or with family videos, to analyze its relationship with clinical outcomes related to different types of language disorders in childhood.

## CONCLUSION

The results demonstrated adequacy regarding the content and construct validity for all signs created in Phase 3 and five of the 12 signs created in Phase 4 of the Enunciative Signs of Language Acquisition (ESLA) instrument. This shows that the ESLA is a promising instrument to assess the language of babies interacting with their mothers.
