# Supplementary material for: Content and construct validation of language acquisition enunciative signs in the second year of life
Source: Codas. 2021 Dec 20;34(2):e20200252. doi: 10.1590/2317-1782/20202020252 (PMC9769415; doi:10.1590/2317-1782/20202020252)
Supplement: Translation

# Translation: Validação de conteúdo e de construto de sinais enunciativos de aquisição da linguagem no segundo ano de vida

**Authors:** Isabela de Moraes Fattore, Anaelena Bragança de Moraes, Anelise Henrich Crestani, Adriano Mendonça Souza, Ana Paula Ramos de Souza

**Affiliations:** 1 Universidade Federal de Santa Maria – UFSM - Santa Maria (RS), Brasil.

**Keywords:** Aquisição da Linguagem, Desenvolvimento Infantil, Validade de Testes, Fatores de Risco, Intervenção Precoce

## Abstract

**Objetivo:**

Analisar a validade de conteúdo e de construto de sinais enunciativos de aquisição da linguagem para crianças de 13 a 24 meses.

**Método:**

Os sinais criados foram submetidos à avaliação de clareza e pertinência por seis juízes experts em aquisição da linguagem na perspectiva enunciativa. A partir do seu julgamento foi produzida uma versão experimental que foi aplicada na análise de vídeos de interações mãe-bebê/examinador, com duração de 15 minutos, de 77 díades mãe-bebê na faixa etária de 13 a 18 meses e 89 díades na faixa etária de 19 a 24 meses. A validação de fidedignidade e consistência interna foi realizada pela análise de 10% da mostra por dois juízes fonoaudiólogos. A validação do construto foi realiza pela análise fatorial efetivada sobre a amostra total. Os dados foram analisados no Statistica 9.1 e PASW 17.0.

**Resultados:**

Todos os sinais foram considerados claros e pertinentes pelos juízes experts. A análise de fidedignidade apontou a concordância quase perfeita (0,8 ≤ Kappa ≥ 1,0) para a maior parte dos sinais. A consistência interna para a Fase 3 apresentou alpha=0,771 considerada alta e Fase 4 apresentou alpha=0,917 limítrofe a muita alta. A análise fatorial da fase 3 revelou 2 fatores, explicando 59,1% da variância total e a fase 4 revelou 1 fator, explicando 75,7%.

**Conclusão:**

Observou-se validade de conteúdo e de construto para cinco dos doze sinais na Fase 3 e para todos sinais na fase 4.

## INTRODUÇÃO

Os Sinais Enunciativos de Aquisição da Linguagem (SEAL), cujo processo de validação de conteúdo e de construção para o primeiro ano de vida já se encontram publicados^(1,2)^, possuem como perspectiva teórica que tanto a contribuição do bebê quanto a do adulto, que sustenta o bebê enunciativamente, são fundamentais no processo de aquisição da linguagem^(3)^. Nessa perspectiva o diálogo é a unidade de análise no processo de avaliação da linguagem^(4,5)^ que permite verificar não apenas se a criança está dominando progressivamente a gramática da língua a qual está exposta, mas também se o ambiente lhe dá condições de inserção no funcionamento de linguagem. Nesse sentido, o SEAL difere de instrumentos tradicionais de triagem como o DENVER II, utilizado na realidade brasileira, que enfoca as produções infantis, sem analisar o contexto ambiental, cujos resultados de pesquisa questionam a sensibilidade e especificidade no primeiro ano de vida^(6)^. Além disso, algumas pesquisas apontam a carência de testes validados para a população brasileira nessa faixa etária^(7,8)^.

O SEAL segue o paradigma indiciário de análise presente em outros instrumentos que avaliam risco ao desenvolvimento^(9)^, que se apresentaram relacionados ao processo de aquisição da linguagem^(10)^. Nesse sentido os sinais são tomados como pistas ou indícios do processo. Assim, a presença de sinais indica que o processo de aquisição está acontecendo conforme o esperado para a faixa etária^(1,2)^, considerando os processos lógicos de emergência de mecanismos enunciativos^(3)^. Já sua ausência indicam que o processo pode estar em risco para um desfecho de atraso na aquisição da linguagem^(1,2)^, na perspectiva da emergência de um sujeito falante^(11)^.

Sabe-se que a validação de um instrumento é fundamental para que sua aplicação seja fidedigna, apresentando confiabilidade e credibilidade^(12)^. Ela dirá se o instrumento mede o que se propõe a medir e se os conceitos abstratos idealizados para o instrumento se converteram em indicadores mensuráveis do fenômeno de interesse. Nesse processo é requerida a análise por especialistas que identificam se os itens são compreensíveis ao profissional que vai utilizá-lo (clareza) e sua pertinência para medição do fenômeno proposto para avaliação^(13)^. Ainda dentro do âmbito da validação de conteúdo, a utilização do instrumento por distintos avaliadores ou no teste e reteste de um mesmo grupo de sujeitos precisa demonstrar fidedignidade e confiabilidade^(14)^, a partir de respostas estáveis e consistentes.

A validação de construção, passo posterior à validação de conteúdo, é fundamental para verificar se o instrumento separa grupos em uma amostra mais ampla, considerando as características investigadas, evidenciando que seus itens de avaliação discriminam essas características, gerando dados precisos, válidos e interpretáveis^(13,14,15)^.

Considerando tais pressupostos, este artigo tem por objetivo apresentar a análise da validação de conteúdo e de construto dos sinais enunciativos de aquisição da linguagem para crianças dos 13 aos 24 meses.

## MÉTODO

Esta pesquisa seguiu as normas éticas obrigatórias para pesquisas em seres humanos – Resolução 466/12 do Conselho Nacional de Saúde – CNS, com projeto aprovado pelo Comitê de Ética da Universidade em que o estudo foi realizado, sob número 18608413.4.0000.5346. Garantiu-se a confidencialidade dos dados, sigilo e privacidade dos sujeitos com a assinatura do Termo de Confidencialidade, assim como o Termo de Consentimento Livre e Esclarecido para os juízes e responsáveis pelos bebês participantes.

A pesquisa trata da validação de conteúdo e de construto de dois instrumentos de análise de linguagem para o segundo ano de vida, denominado neste estudo por Sinais Enunciativos de Aquisição da Linguagem (SEAL). Os dois instrumentos dividem-se em Fase 3, o qual corresponde aos sinais pertinentes à faixa etária de 13 a 18 meses, e Fase 4 que corresponde aos sinais pertinentes à faixa etária de 19 a 24 meses.

Uma versão inicial dos instrumentos foi proposta considerando os mecanismos e estratégias enunciativas^(3)^ e a experiência clínica com bebês que tiveram atraso na aquisição da linguagem. Os sinais elaborados foram analisados por seis juízes experts em aquisição da linguagem em uma perspectiva enunciativa e, após contribuições dos mesmos para aprimoramento dos sinais, foram julgados pelos mesmos juízes quanto à clareza e pertinência dos sinais. Para tanto, eles utilizaram uma escala de *Likert* de cinco pontos: 1- discordo totalmente, 2-discordo parcialmente, 3- não concordo nem discordo, 4- concordo parcialmente, 5- concordo totalmente para cada sinal proposto. Os sinais que atingiram percentual igual ou superior a 70%^(16)^, de atribuição no item 4-concordo parcialmente e/ou 5-concordo totalmente, permaneceram nos instrumentos para constituir a versão experimental.

Para análise das interações mãe-bebê foram gravados os vídeos de interações de 15 minutos, de 166 bebês nas faixas etárias de 13 a 24 meses e suas mães, que foram captados em uma unidade básica de saúde próxima à universidade, durante o teste do pezinho, que serviu também como parte do processo de triagem dos bebês. Eles também tinham o teste da orelhinha e foram encaminhados para avaliação do potencial cortical para eliminar bebês algum tipo de deficiência auditiva. O grupo de pesquisadores contou com equipe interdisciplinar de fonoaudiólogos, psicólogos, fisioterapeutas e terapeutas ocupacionais, porque apenas bebês nascidos a termo, sem comprometimento físico, auditivo e visual foram incluídos na pesquisa. Além dos testes objetivos já mencionados, os bebês foram observados quanto ao desenvolvimento psicomotor, físico e intelectual por esta equipe. Também as mães não poderiam ter distúrbios psiquiátricos evidentes o que foi observado pelas psicólogas em entrevista inicial que versou sobre aspectos obstétricos, sociodemográficos e psicossiciais do bebê e sua família. Assim, foram excluídos bebês com comprometimento neurológico, malformações e síndromes, prematuros, deficientes auditivos ou visuais. Diante de qualquer dúvida em relação à mãe ou ao bebê, eles foram encaminhados para avaliações mais detalhadas e descartados da pesquisa.

As filmagens foram realizadas em ambiente confortável, com boa iluminação e ventilação, com o bebê devidamente higienizado e alimentado, sem dor ou desconforto. Para efetivação das filmagens foi disponibilizada uma caixa de brinquedos com miniaturas para exploração pela mãe e pela criança, convidadas a permanecer em um tapete de EVA, com uma câmera digital postada sobre cavalete há dois metros de distância que pegava o reflexo da mãe e do bebê no espelho colocado junto à parede atrás do tapete, e outra há um metro de distância que filmava a dupla em ângulo lateral. Os primeiros dez minutos apenas as interações das mães e bebês foram filmadas. Nos últimos cinco minutos o pesquisador entrou e interagiu com o bebê e a mãe para observação de um sinal que envolvia a interação com interlocutor distinto da mãe. Além de ser uma forma de fazer solicitações no diálogo que favorecessem a emergência de protopalavras e palavras, quando estas não apareciam na interação com a mãe.

Após a coleta, as filmagens foram armazenadas em HD para visualização dos pesquisadores e análise dos sinais cujas respostas eram dicotômicas, do tipo sim para presença do sinal e não para ausência do sinal. No caso dos sinais referentes às ações do adulto que versavam sobre mais de uma ação do mesmo (Sinal 7 da fase 3 e Sinal 5 da fase 4) eles foram considerados presentes apenas quando as ações eram realizadas. Por exemplo, se a mãe fornecia interpretações não sintonizadas ao que a criança claramente manifestava (sinal 7 fase 3) ele estaria ausente. No caso do Sinal 5 da fase 4 se a mãe corrigia a fala infantil qualquer uma das três opões observadas (pedido neutro de reparo, repetição correta da fala infantil ou oferta do item correto) permitiu considerar que o sinal estava presente.

Para verificar a fidedignidade, analisando a aplicabilidade e a estabilidade do instrumento, utilizou-se o método entre avaliadores, em que dois fonoaudiólogos analisaram os vídeos de 10% da amostra de modo individual e os resultados de sua análise foi comparado para determinar a concordância entre as respostas, calculando-se o coeficiente de Kappa. Para a análise da Consistência Interna, foi utilizado o teste de Kuder-Richardson (K-R), em função de as respostas serem dicotômicas.

A seguir os sinais foram aplicados em toda a amostra. Quando algum dos sinais estivesse ausente da análise de 10% e se confirmasse esta ausência na análise da amostra total do estudo, ele seria eliminado da análise fatorial

A partir da versão instrumental obtida com essa análise de fidedignidade e consistência interna, a amostra final contou com ao menos 10 díades para cada sinal a ser observado para gerar soluções fatoriais na análise multivariada^(13,14,15)^, considerando nível de significância de 5%. A amostra considerada nesta fase, foi de 77 díades para fase 3, e 89 díades para a fase 4, conforme já relatado. Para a análise fatorial foi utilizado o coeficiente de Kaiser-Meyer-Olkin (KMO) e o teste de esfericidade de Bartlett.

Os dados coletados foram armazenados em uma planilha eletrônica no excel e convertidos para programas computacionais STATISTICA 9.1, PASW 17.0 e Software-R. A análise quantitativa dos dados foi efetuada por meio da estatística descritiva e inferencial, utilizando testes específicos a cada etapa, já mencionados.

## RESULTADOS

No Quadro 1 estão os sinais enunciativos de aquisição da linguagem construídos pelas pesquisadoras e apresentados aos juízes experts para análise de conteúdo.

**Quadro 1:** Versão inicial dos sinais enunciativos de aquisição da linguagem

Fase 3	*Descrição*
S1	A criança nomeia de modo espontâneo e inteligível ao adulto interlocutor, objetos que estão ausentes no contexto.
S2	A criança nomeia objetos que estão ausentes no contexto de modo espontâneo, mas não inteligível ao adulto interlocutor, buscando na prosódia uma forma de ser compreendida.
S3	A criança nomeia de modo espontâneo e inteligível ao adulto interlocutor, objetos, pessoas, ações, que estão presentes no contexto enunciativo.
S4	A criança faz gestos para tentar fazer-se entender quando o adulto interlocutor não a compreende.
S5	A criança repete o dizer do adulto interlocutor como forma de organizar ou reorganizar sua enunciação, por exemplo, aprimorando a forma sintática, ou fonológica, ou a escolha do item lexical ou mesmo acentuando algum item prosodicamente.
S6	A criança conversa com diferentes interlocutores adultos (pai, mãe, examinador).
S7	O adulto interlocutor atribui um sentido possível às produções verbais da criança de modo sintonizado.
Fase 4	*Descrição*
S1	A criança solicita objetos e/ou pede esclarecimentos ao adulto interlocutor, marcando sua posição como locutor.
S2	A criança utiliza formas fonêmicas distintas para veicular sentidos diferentes em sua enunciação (ao menos dois modos e dois pontos articulatórios em produções mono ou dissílabas- por exemplo ma, da, ta, na, pa, ba)
S3	A criança utiliza distintas formas (palavras) para veicular sentidos diferentes em sua enunciação
S4	A criança combina palavras, na forma direta ou inversa, para veicular sentidos diferentes.
S5	Quando a criança apresenta produções verbais distintas da fala adulta, o adulto interlocutor reage fazendo um pedido de reparo neutro (o que) ou repetindo corretamente a fala infantil ou oferecendo palavra mais adequada.
S6	A criança faz comentários ou qualificações de situações (por exemplo, está pesado, bom, ruim,tá,aqui,ele tá lá).
S7	A criança demonstra refletir sobre os acontecimentos por meio da produção de enunciados de mais de uma palavra (por exemplo: Não sei se ele caiu, Será que ele vem?)
S8	A criança reformula seu dizer diante do não entendimento do adulto interlocutor e marca quando não entende o enunciado do interlocutor
S9	A criança se marca discursivamente como um “eu” (com o uso do pronome ou na flexão do verbo).
S10	A criança se marca pela forma nominal ou por formas pronominais que não seja o “eu” no discurso (por exemplo, nós, teu, meu, próprio nome)
S11	A criança insere um dizer anterior (seu ou de outros) ou projeta um dizer futuro em seu discurso
S12	A criança simula diálogos em seu discurso, constituindo situações fictícias.

Na Tabela 1 estão os resultados da análise de clareza e pertinência realizada pelos juízes experts.

**Tabela 1:** Análise de clareza e pertinência dos sinais fases 3 e 4 por juízes experts

sinais	Análise de clareza								Análise de Pertinência							
Fase3	J1	J2	J3	J4	J5	J6	%CT	%CP+CT	J1	J2	J3	J4	J5	J6	%CT	%CP+CT
S1	4	5	5	4	5	5	66,7	100	4	5	5	5	5	5	83,3	100
S2	5	5	5	4	5	5	83,3	100	4	5	5	5	5	5	83,3	100
S3	4	5	5	4	5	5	66,7	100	4	5	5	5	5	5	83,3	100
S4	5	5	5	5	5	5	100	100	5	5	5	5	5	5	100	100
S5	4	5	5	5	5	5	83,3	100	4	5	5	5	5	4	66,7	100
S6	5	5	5	5	5	5	100,0	100	5	5	5	5	5	5	100	100
S7	4	5	4	4	4	5	33,3	100	4	5	5	5	5	5	83,3	100
Fase 4	5	5	5	3	5	5	83,3	83,3	5	5	5	5	5	5	100	100
S1	5	5	4	5	5	5	83,3	100	5	5	4	5	5	5	100	100
S2	5	5	5	5	5	5	100	100	5	5	5	5	5	5	100	100
S3	5	5	5	4	5	5	83,3	100	5	5	5	5	5	5	100	100
S4	5	5	5	5	5	5	100	100	5	5	5	5	5	1	83,3	83,3
S5	4	5	5	4	5	5	66,7	100	4	5	5	5	5	5	83,3	100
S6	5	5	5	4	5	5	83,3	100	4	5	5	5	5	5	83,3	100
S7	6	5	5	4	5	5	83,3	100	4	5	5	5	5	5	83,3	100
S8	5	5	5	5	5	5	100	100	5	5	5	5	5	5	100	100
S9	5	5	5	5	5	5	100	100	5	5	5	5	5	1	83,3	83,3
S10	5	5	4	5	5	5	83,33	100	5	5	5	5	5	5	100	100
S11	5	5	5	1	5	5	83,33	83,33	5	5	5	4	5	5	83,3	100
S12	5	5	5	3	4	5	66,7	83,33	5	5	5	5	5	5	100	100

Legenda: J = Juiz; %CP = percentual concordância parcial; %CT = percentual concordância total; S = Sinal; 1 = discordo totalmente; 3 = não concordo nem discordo; 4 = concordo parcialmente; 5 = concordo totalmente

O critério inicial para permanência do sinal partiu do percentual de juízes que concordaram parcialmente e/ou totalmente com cada sinal. Percentuais de concordância inferiores ou iguais a 70%^(16)^ foram avaliados quanto a sua alteração ou exclusão pelas pesquisadoras. Como se pode observar na Tabela 1, não houve percentual inferior a 83,3% quando somadas os níveis 4 (concordância parcial) e 5 (concordância total) da escala de likert tanto em relação à clareza quanto à pertinência. A partir dessa análise foi possível construir uma versão experimental do SEAL, sintetizada no Quadro 2.

**Quadro 2:** Versão experimental do SEAL

Fase 3	*Descrição*
S1	A criança nomeia de modo espontâneo e inteligível ao adulto interlocutor, objetos que estão ausentes no contexto.
S2	A criança nomeia objetos que estão ausentes no contexto de modo espontâneo, mas não inteligível ao adulto interlocutor, buscando na prosódia uma forma de ser compreendida.
S3	A criança nomeia de modo espontâneo e inteligível ao adulto interlocutor, objetos, pessoas, ações, que estão presentes no contexto enunciativo.
S4	A criança faz gestos para tentar fazer-se entender quando o adulto interlocutor não a compreende.
S5	A criança repete o dizer do adulto interlocutor como forma de organizar ou reorganizar sua enunciação, por exemplo, aprimorando a forma sintática, ou fonológica, ou a escolha do item lexical ou mesmo acentuando algum item prosodicamente.
S6	A criança conversa com diferentes interlocutores adultos (pai, mãe, examinador).
S7	O adulto interlocutor atribui um sentido possível às produções verbais da criança de modo sintonizado.
Fase 4	*Descrição*
S1	A criança solicita objetos e/ou pede esclarecimentos ao adulto interlocutor, marcando sua posição como locutor.
S2	A criança utiliza formas fonêmicas distintas para veicular sentidos diferentes em sua enunciação (ao menos dois modos e pontos articulatórios- por exemplo ma, da, ta, na, pa, ba)
S3	A criança utiliza distintas formas (palavras) para veicular sentidos diferentes em sua enunciação (possui variedade lexical)
S4	A criança combina palavras, na forma direta ou inversa, para veicular sentidos diferentes.
S5	Quando a criança apresenta produções verbais distintas da fala adulta, o adulto interlocutor reage fazendo um pedido de reparo neutro (o que) ou repetindo corretamente a fala infantil.

Legenda: S = sinal. Fonte: Autoras

Quanto à fidedignidade, na atribuição de sinais na análise dos vídeos por dois juízes, para a Fase 3 (13 a 17 meses completos) e Fase 4 (18 a 24 meses completos), os resultados obtidos estão expostos na Tabela 2. Cabe destacar que, na fase 4, os sinais 6, 7,8,9,10, 11 e 12 não foram encontrados na amostra de 10% nem na amostra total, o que indicou sua eliminação. Por isso na Tabela 2, são apresentadas apenas as concordâncias dos sinais 1 a 7 na fase 3 e 1 a 5 na fase 4, os sinais encontrados pelas juízas em ambas amostras analisadas.

**Tabela 2:** Concordância entre avaliadores do SEAL Fase 3 e Fase 4

**Sinais SEAL Fase3**	**% concordância**	**Coeficiente Kappa**	p-valor^*^
S1	100	1,00	-
S2	98,7	-	-
S3	88,3	0,746	-
S4	72,7	0,357	0,001
S5	92,2	0,834	-
S6	98,7	0,971	-
S7	92,2	0,798	-
**Sinais SEAL Fase 4**			
S1	96,6	0,916	0,000
S2	98,9	0,967	0,000
S3	98,9	0,970	0,000
S4	97,8	0,954	0,000
S5	97,8	0,947	0,000

* Significativo pela análise de Concordância Kappa com nível de significância de 5%

Legenda: SEAL = Sinais enunciativos de Aquisição da Linguagem; % = porcentagem

Na Fase 3, para o Sinal 2, Kappa não foi calculado por não apresentar variabilidade na avaliação dos sinais por ao menos um dos juízes. Os sinais 3 e 7 apresentaram concordância substancial (0,6 ≤ Kappa ≥ 0,79). O sinal 4 apresentou concordância fraca substancial (0,2 ≤ Kappa ≥ 0,3). A concordância dos juízes foi quase perfeita (0,8 ≤ Kappa ≥ 1,0) nos sinais 1, 5 e 6 da Fase 3 e em todos sinais da Fase 4.

O método de Consistência Interna foi aplicado nos dois instrumentos, após realizada análise da concordância em relação aos sinais. Utilizou-se o coeficiente alfa de Cronbach na amostra total, ou seja, Fase 3=77 díades e Fase 4=89 díades, analisando cada sinal individualmente. Para a Fase 3, obteve-se alpha = 0,771 e para Fase 4, alpha=0,917.

A seguir foi realizada a análise fatorial da fase 3, conforme pode ser visto na Figura 1.

**Figura 1:** Fase 3 – Distribuição de Sinais – Fator 1- Sinais 5,3,6,7; Fator 2 - Sinal 2

Fonte: Análise Multivariada. Legenda: s = sinal

Como pressuposto para a realização da análise fatorial do instrumento da Fase 3 (13 a 17 meses) obteve-se o coeficiente Kaiser-Meyer-Olkin (KMO = 0,78), considerado moderado para indicar adequação do instrumento à análise fatorial, ou seja, a análise fatorial é apropriada, e o teste de esfericidade de Bartlett (159,7; p < 0,01) cuja significância indica que as variáveis estão correlacionadas entre si. Assim, a análise fatorial da Fase 3 revelou a existência de 2 fatores, os quais explicaram 59,1% da variância total. A análise com Rotação Varimax mostrou o fator 1 com quatro itens e o fator 2 com um item. Ao realizar a Análise Fatorial com rotação Varimax observou-se no fator 1, quatro variáveis de maior relevância, na seguinte ordem: sinais 5, 3, 6 e 7 e o fator 2 com uma variável de maior relevância, o sinal 2.

O fator 1 determinado por um autovalor λ = 3,1 e correspondendo a uma variância explicada de 46,6%, representa *“ a criança sustentada pelo adulto e com possibilidades de progressão na aquisição lexical”.* O fator 2 determinado por um autovalor λ = 1,1e correspondendo a uma variância explicada de 15,5%, representa *“a intenção comunicativa sendo um fator para o diagnóstico diferencial”.*

A análise fatorial da Fase 4 revelou a existência de um único fator, que explicou 75,7% da variância total. A análise sem Rotação Varimax – já que tem-se apenas um fator, mostrou o fator 1 com cinco itens na seguinte ordem de relevância: Sinal 3 (0,938) – A criança utiliza distintas formas (palavras) para veicular sentidos diferentes em sua enunciação; Sinal 5 (0,870) – Quando a criança apresenta produções verbais distintas da fala adulta, o adulto interlocutor reage fazendo um pedido de reparo neutro (o que) ou repetindo corretamente a fala infantil ou oferecendo item lexical correto; Sinal 1 – (0,864)- A criança solicita objetos e/ou pede esclarecimentos ao adulto interlocutor, marcando sua posição como locutor; Sinal 2(0,852) - A criança utiliza formas fonêmicas distintas para veicular sentidos diferentes em sua enunciação (ao menos dois pontos articulatórios – labial e alveolar- e duas classes sonoras consonantais distintas – ao menos nasais e plosivas, por exemplo ma, da, pa, etc); e sinal 4 (0,821)-A criança combina palavras na forma direta ou inversa, para veicular sentidos diferentes.

## DISCUSSÃO

Considerando a análise pelos *experts* e a concordância entre os avaliadores, houve uma aprovação razoável do instrumento. O único sinal que apresentou concordância fraca substancial (0,2 ≤ Kappa ≥ 0,3) entre os avaliadores foi o sinal 4 da fase 3. Na análise fatorial realizada, constatou-se que este sinal não ficou incluso em nenhum fator dos dois encontrados na fase 3 do instrumento, não se correlacionado com nenhum dos demais sinais, embora tenha sido considerado claro e pertinente pelos experts.

Na análise fatorial, o sinal 5 apresentou um peso maior no fator 1, o que demonstra a importância da sustentação enunciativa no processo de aquisição^(3)^, ou seja, se a criança se socorre do interlocutor para a construção gramatical. Essa sustentação enunciativa é denominada na literatura internacional de *scaffolding* ^(16)^.

Ainda na fase 3, percebe-se que a concordância existente entre os juízes na aplicação dos sinais, diante do coeficiente Kappa, indica concordância excelente nos sinais 1, 5,6 e 7; concordância suficientemente boa no sinal 3 e concordância pobre o sinal 4, o que se confirma na ausência de força discriminatória deste sinal na análise fatorial e maior poder distintivo nos outros quatro sinais.

Quanto ao sinal 2 da Fase 3, a presença deste sinal nas crianças avaliadas prevê que a criança possa estar usando recursos prosódicos para escapar a um menor domínio de gestos articulatórios. Se junto com este sinal se evidencia a ausência do sinal 2 na fase 4, pode-se ficar alerta para um risco para a realização vocal da língua em termos benvenisteanos, o que deve ser objeto de observação no decorrer da evolução do balbucio para as protopalavras, aspectos básicos na organização das praxias de fala necessárias à produção inicial dos vocábulos da língua. Um estudo em aquisição da linguagem indica a relação entre a fala e o gesto corporal^(17)^, o que permite ressaltar que o Sinal 2 da Fase 3 pode ser tomado como evidência de empenho em se fazer entender pelo interlocutor, principalmente se estiver presente em conjunto com a nomeação inteligível na mesma fase (sinais 1 e 3) e com a presença do sinal 2 da fase 4.

Quanto à consistência interna, observou-se na fase 3, o valor de alpha = 0,771, que sugere que os sinais apresentados não se sobrepõem na avaliação. Essa medida de confiabilidade indica o grau de correlação entre os itens, representando uma mensuração da confiabilidade. Assim, pode-se dizer que os itens estão correlacionados entre si, mas não estão mensurando a mesma coisa, considerando o valor de alpha encontrado. Diferentemente do que foi evidenciado na fase 4, na fase 3 houve a presença de dois fatores.

O fator 1 ficou composto pelos sinais 5, 3, 6 e 7, em ordem de relevância. Os sinais 5, 3 e 6 são esperados no curso de uma aquisição típica considerando que a criança estaria apta para utilizar-se de seu conhecimento lexical, aprimorando-o com distintos interlocutores. O sinal 7 demonstra o papel do adulto na sustentação da criança no diálogo, de forma a mantê-la empenhada nessa construção com o outro. O adulto atribui sentido ao dizer da criança, mesmo que a forma não esteja facilmente compreensível, mas observado o contexto, é passível de entendimento. Quando ausentes, os sinais do fator 1, já evidencia-se, precocemente, um conjunto de condições enunciativas que evidenciam que a criança não entrou no segundo mecanismo enunciativo, relacionado à possibilidade de passar da referência mostrada à falada, ou seja, podem evidenciar que a criança pode ter sua posição como falante estremecida no ambiente social, havendo dúvidas da suposição de falante da língua.

O fator 2, na fase 3, composto somente pelo sinal 2, sugere o que pode evidenciar as dificuldades de inteligibilidade de fala de uma criança compensadas pela prosódia, ou seja, que a realização vocal da língua^(3)^ pode estar empobrecida e que o desfecho clínico poderia ser ou não uma patologia de fala após diagnosticada mais tardiamente. Novamente, cabe a observação que estudos com desfechos clínicos que poderão ou não confirmar tal hipótese, serão possíveis após a validação de critério destes sinais. Essa dificuldade evidenciada entre 18 e 24 meses pode levar o adulto a ter dificuldades no reconhecimento do signo, um dos indicadores de risco encontrados por Verly e Freire^(11)^ ao analisarem casos de atraso na aquisição da linguagem.

Na Fase 4, a concordância entre avaliadores foi excelente para todos os itens propostos. Na consistência interna, realizada pelo coeficiente alfa de Cronbach, o alpha na fase 4 ficou um pouco acima de 0,90, indicando possível redundância ou duplicação dos itens quanto ao que estão medindo, ou seja, todos os sinais tem o mesmo valor diagnóstico. Esse dado confirma-se na análise fatorial, na qual, constatou-se um único fator nesta fase.

Em uma leitura enunciativa, quanto mais capaz de estabelecer a co-referência com o ouvinte, por meio de itens lexicais variados, mais a criança demonstra domínio semiótico da língua e também ampliação de estratégias do segundo mecanismo enunciativo^(3)^. Além de a criança apresentar um léxico ampliado, ela começa a construir frases para poder enunciar de modo mais complexo durante a interlocução. Assim, quanto maior o léxico, maiores as facilidades na compreensão e produção linguísticas, o que Silva^(3)^ afirma ampliar-se de modo importante em funções como interrogação, intimação, bem como o assumir posições discursivas variadas no terceiro mecanismo enunciativo.

Na fase 4, já se evidenciam se há os reparos ou a repetição correta da fala infantil, por parte do adulto, conforme descrito no sinal 5. A relevância do sinal 5 (0,870) diz respeito a quanto o adulto demonstra estar ou não atento ao que a criança produz e o quanto essa produção se distancia da forma adulta, podendo receber reparos por parte do adulto.

O sinal 3 da fase 4 (0,938), considerado o mais relevante de todos que compõem o fator, evidencia que a criança está utilizando da variedade lexical que é adequada para a sua faixa etária, não se restringindo apenas itens lexicais repetidos ou formas neutras como pronomes demonstrativos. Das protopalavras, a criança passa a produzir as primeiras palavras, que já demonstram uma variação de acentuação e com função de comunicar algo. Diante do domínio lexical que a criança vai adquirindo, sobretudo de verbos, o aparecimento das frases começa a ser frequente, já em idade um pouco mais avançada^(3)^.

Ainda na fase 4, o sinal 1 (0,864) já indica a presença da criança no terceiro mecanismo enunciativo^(3)^, no qual ocorre a instauração do sujeito na língua-discurso, ou seja, a criança já está inserida no aparelho das funções, tomando iniciativa, para intimar e interrogar, marcando seu lugar como sujeito no diálogo com o outro. Além de um falante da língua, marca-se de diferentes formas no discurso^(18)^.

Quanto ao sinal 2 da fase 4 (0,852), relaciona-se a maior sensibilidade para detectar precocemente ausência de risco para a realização vocal da língua, ou seja, realização do gesto articulatório, conforme já discutido. Ele busca ver a capacidade de variação fonética na realização do gesto articulatório. Por isso, propõe-se que os sinais permitam acompanhar o curso de produção das praxias orais e tomar algumas medidas de orientação familiar e/ou intervenção a tempo de modo a aproveitar a plasticidade cerebral, visto que quanto mais precoce a intervenção melhores são as chances de superação dos sintomas. Isso motiva estudos neurocientíficos de análise de processamento sensorial precoce nos bebês^(19)^, bem como fatores socioeconômicos que podem interferir no desenvolvimento cerebral, sobretudo educação parental^(20)^. É possível utilizar a sustentação enunciativa^(3-5)^ e estratégias prosódicas de ancoragem musical da gestualidade corporal e oral para facilitar a evolução dos bebês com risco psíquico e à aquisição da linguagem^(21)^. A música foi especialmente importante no relato de caso clínico de tratamento de apraxia de fala em criança de 5 anos. Ela permitiu uma abordagem significativa na articulação entre ouvir e escutar e falar e dizer^(22)^. Cabe destacar, no entanto, que risco não é diagnóstico e que ele deve ser tomado de um modo a oportunizar estratégias promocionais da aquisição sem criar fantasmas no imaginário parental. Por isso, acredita-se na musicalização como estratégia interessante de intervenção para crianças entre 18 e 24 meses.

Um aspecto a ser ressaltado é que tanto os sinais infantis quanto os sinais do adulto interlocutor do bebê, no caso desta pesquisa sempre a mãe biológica, evidenciaram potência na identificação de diferenças na amostra. Isso indica que a sustentação enunciativa^(3)^ é crucial no processo de aquisição da linguagem infantil, dado o caráter intersubjetivo do funcionamento de linguagem^(18)^.

O estudo apresentado teve como limitações a variação do retorno dos casos para a continuidade das avaliações, o que restringiu a obtenção de amostra maior.

É importante ressaltar que o SEAL ainda possui um longo caminho para sua validação, pois é necessário estabelecer os critérios para emergência dos sinais a cada faixa etária, bem como realizar estudos de coorte populacionais ou com vídeos familiares para analisar a relação do mesmo com desfechos clínicos relacionados aos distintos tipos de distúrbios de linguagem na infância.

## CONCLUSÃO

Os resultados demonstraram adequação quanto à validade de conteúdo e de construto para todos sinais criados na fase 3 e cinco dos doze sinais criados para fase 4. Isso evidencia que o SEAL é um instrumento promissor para avaliação da linguagem de bebês em interação com suas mães.
